# A little more conversation, a little more action, please: the carbon footprint of travelling to conferences of the European Health Psychology Society

**DOI:** 10.1080/21642850.2024.2447454

**Published:** 2025-01-02

**Authors:** Lisa M. Warner, Ruvn Fleiner, Gudrun Sproesser, James A. Green, Lucia Rehackova, Jennifer Inauen, Vera Araújo-Soares, Claudia Teran-Escobar

**Affiliations:** a Department of Psychology, MSB Medical School Berlin, Berlin, Germany; b Health Psychology, Johannes Kepler University Linz, Linz, Austria; c School of Allied Health and Health Research Institute, University of Limerick, Limerick, Ireland; d Faculty of Health and Life Sciences, Department of Nursing, Midwifery, and Health, Northumbria University, Newcastle upon Tyne, UK; e Institute of Psychology, University of Bern, Bern, Switzerland; f Center for Preventive Medicine and Digital Health (CPD), Medical Faculty Mannheim, Heidelberg University, Heidelberg, Germany; g Department of Psychology, University Paris Nanterre, Nanterre, France

**Keywords:** CO_2_ equivalent, greenhouse gas emissions, air travel, sustainable mobility, flying

## Abstract

**Introduction:**

The environmental impact of on-site conferences, with air travel as the primary contributor to greenhouse gas emissions, has prompted a surge in research in recent years. The objective of this report is to raise awareness and stimulate transformation in the organisation of meetings of the European Health Psychology Society (EHPS).

**Methods:**

We conducted estimations of travel-related CO_2_eq emissions of EHPS conferences in 2019, 2022, and 2023, and performed projections for 2024 and 2025. Additionally, we developed hypothetical scenarios for selected European cities as centroids for future conferences.

**Results:**

EHPS conferences with an online option result in significant reductions in CO_2_eq emissions when compared to on-site only conferences. The selected European locations of these conferences enable more delegates to choose alternative forms of transportation instead of flying, such as trains, cars or buses, and consequently lead to significantly lower CO_2_eq emissions.

**Discussion:**

The principal avenues for curbing travel-related emissions while maintaining on-site attendance are the provision of hybrid conferences with enhanced online participation and the optimisation of venue locations.

## Introduction

The European Health Psychology Society (EHPS) aims to promote behaviour change for human and planetary health. It has: (a) established a Special Interest Group on ‘Equity, Global Health and Sustainability'; (b) from 2019, mapped conference presentations to United Nations Sustainable Development Goals; (c) introduced a ‘Climate Change and Sustainability’ track to its annual conferences (d) and launched initial sustainability initiatives during its conferences (Warner et al., 2022, 2023).

Climate change already manifests in rising sea levels and increasing intensity and frequency of weather events, e.g. heat waves, wildfires, desertification, storms and floods (IPCC, 2023). Reducing CO_2_ (and other greenhouse gas emissions that are fuelling climate change) to limit global warming to 1.5°C or 2°C requires major collective and coordinated effort to change individual (e.g. daily mobility, holiday air travel), collective and professional behaviour, such as business air travel (IPCC, 2023; Whitmarsh et al., 2021). To raise scientists’ – our own – awareness, this report estimates the travel emissions from EHPS conferences.

Scientists emit many more greenhouse gases due to travel activities than non-scientists (Stroud & Feeley, 2015; Whitmarsh et al., 2020). A scientist’s air travel for one intercontinental conference can result in two to five tonnes of CO_2_eq.^1^ This is well above the annual limit of 1.5–2.3 tonnes of CO_2_eq per person required to mitigate climate change and comply with the Paris Agreement to limit warming to the 1.5°C target by 2030 (Atmosfair, n.d.; Gore, 2021). For on-site conferences, air travel is by far the largest source of emissions, accounting for 80–96% of the total conference-related CO_2_eq emissions (followed by approx. 10% accommodation, 3% food; Hischier & Hilty, 2002; Leochico et al., 2021; Neugebauer et al., 2020; Tao et al., 2021; Wadud et al., 2024; Zotova et al., 2020).

Estimates show that online or hybrid conferences (on-site + online option) would drastically reduce emissions (Cavallin Toscani et al., 2023; Jäckle, 2021). However, scientists' willingness to adapt their travel behaviour can still be increased (Barron et al., 2020; Pellarin et al., 2023; Teran-Escobar et al., 2024; Whitmarsh et al., 2020).

An important first step for scientific societies and event organisers is to assess the carbon footprint of their meetings, which enables discussing ways of reducing it. The objectives of this report are, therefore, to:

1. estimate travel-related CO_2_eq emissions caused by on-site and hybrid EHPS conferences in 2019, 2022, and 2023,
2. compare the travel emissions of the different conference formats (on-site only: Dubrovnik 2019; hybrid: Bratislava 2022, Bremen 2023),
3. model the impact of EHPS conference locations on travel emissions, by making projections for conferences in Cascais (Portugal, 2024) and Groningen (Netherlands, 2025)
4. model the impact of EHPS conferences held in selected European cities (Amsterdam, Paris, Brussels, Frankfurt, London) on travel emissions.

Our hypotheses are that:

1. hybrid conferences result in significantly lower CO_2_eq travel emissions than on-site only conferences.
2. cities that minimise travel-distance for most participants will have lower estimated CO_2_eq travel emissions.

## Methods

Anonymised affiliation data for each participant registered for EHPS conferences in 2019, 2022, and 2023 were obtained from the conference registration system. By filling in the registration form, each participant agreed that their anonymised location data would be analysed for statistical purposes (the analyses received an ethics exemption letter by the ethics commission of the MSB Medical School Berlin #MSB-2024/205). Three variables were extracted: (a) country of affiliation, (b) city of affiliation, (c) mode of participation (on-site versus online). We assumed that participants’ primary institutional affiliation represented their departure city. Travel emissions were based on adapted estimates (Desiere, 2016) published by Klöwer et al. (2020; see methods part in Supplement 1). The European cities used as hypothetical comparisons (Amsterdam, Paris, Brussels, Frankfurt, London) have been chosen to minimise the distance from the affiliation data to cities with very good international travel connections.

From 2022 on, the EHPS has offered hybrid conferences, requiring consideration of online participants' emissions (energy use etc.). The emission model developed by Faber (2021) was used to calculate minimum (i.e. 27 kgCO_2_eq/conference for participants attending 2 h/day) and maximum (i.e. 45 kgCO_2_eq/conference for participants attending 8 h/day) online attendance emissions (details, see Supplement 2 by Faber, 2021). Each online participant was assigned an individual emission CO_2_eq value within this range, assuming attendance rates between 2 and 8 h per day x 3.5 days. We simulate scenarios for Cascais and Groningen assuming the same number of participants as in Bremen, participating the same way (i.e. on-site/online).

## Ethics statement

- Institutional Review Board Statement: The study was conducted in accordance with the Declaration of Helsinki and was approved by an Institutional Review Board/Ethics committee. See details under Methods.
- The study received an exemption from an Institutional Review Board/Ethics committee; See details under Methods.

## Results

**Figure 1.**
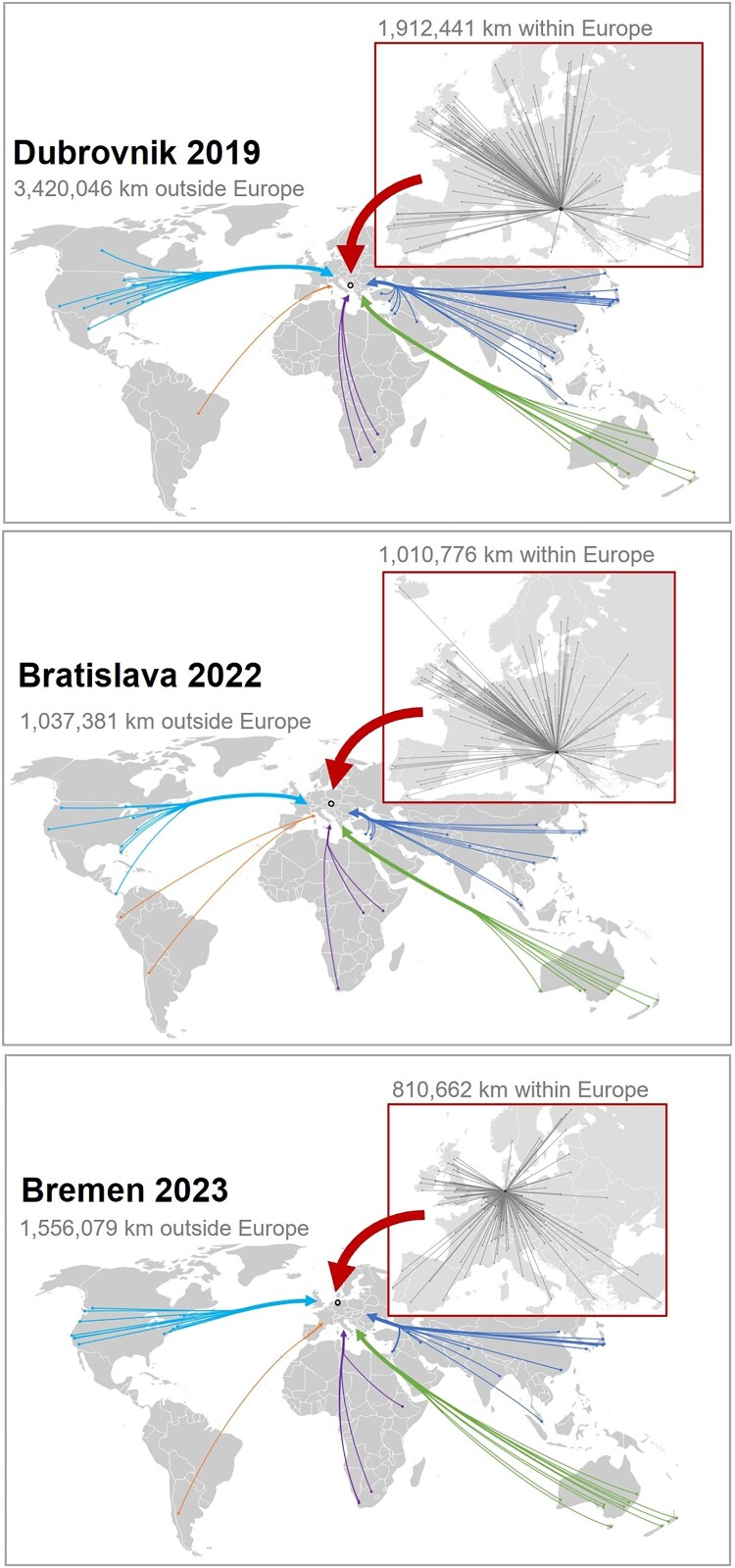
Sum of distances travelled to EHPS conference locations in 2019–2023, by travel distances within and outside of Europe (on-site participants only). Source: World Map Original by Vardion, svg-ified by Simon Eugster on Wikipedia (https://de.wikipedia.org/wiki/Datei:BlankMap-World_gray.svg); Map of Europe on Wikipedia (https://upload.wikimedia.org/wikipedia/commons/0/0c/Blank_map_Europe_with_borders.png).

Total distances travelled to each of the past three EHPS conferences within Europe and from overseas are illustrated in Figure 1 and Table 1.

**Table 1.** Estimates of CO_2_eq emissions per participant and conference by mode of participation (on-site only travel emission / online only IT emissions).

	Dubrovnik 2019	Bratislava 2022			Bremen 2023			Projection to Cascais 2024			Projection to Groningen 2025		
	on site only	hybrid (on site + online)	on site only	online only	hybrid (on site + online)	on site only	online only	hybrid (on site + online)	on site only	online only	hybrid (on site + online)	on site only	online only
*N* of participants registered	857	696	526	170	795	729	66	795	729	66	795	729	66
*n* from Europe (%)	674 (78.6%)	595 (85.5%)	466 (88.6%)	129 (75.9%)	697 (87.7%)	645 (88.5%)	52 (78.8%)	697 (87.7%)	645 (88.5%)	52 (78.8%)	697 (87.7%)	645 (88.5%)	52 (78.8%)
Total km travelled by all participants	5,332,488	–	2,048,158	–	–	2,366,742	–	–	4,232,367	–	–	2,358,572	–
*M* (*SD*) in km roundtrip per participant	6222 (7323)	–	3894 (5765)	–	–	3247 (6370)	–	–	5806 (6328)	–	–	3235 (6412)	–
Number of return trips to the moon	13.87	–	5.33	–	–	6.16	–	–	11.01	–	–	6.14	–
Number of trips around earth	133.06	–	51.11	–	–	59.06	–	–	105.61	–	–	58.85	–
Total CO2eq in t for conference	1422	507	503	4	593	592	2	1123	1122	2	576	574	2
*M* (*SD*) CO2eq in t per participant	1.66 (2.23)	0.73 (1.55)	0.96 (1.72)	0.026 (0.004)	0.75 (1.84)	0.81 (1.91)	0.025 (0.004)	1.41 (1.90)	1.54 (1.93)	0.025 (0.004)	0.72 (1.86)	0.79 (1.93)	0.025 (0.004)
CO2eq in # of av. emissions of an EU household per year	142.19	50.71	50.28	0.43	59.34	59.17	0.17	112.36	112.19	0.17	57.59	57.42	0.17
CO2eq in tennis courts of melted artic sea ice	16.35	5.83	5.78	0.05	6.82	6.80	0.02	12.92	12.90	0.02	6.62	6.60	0.02

Note: For Cascais and Groningen the number of total, online and on-site participants is based on Bremen numbers.

For a better understanding of the large travel-distances Table 1 shows equivalent numbers of journeys around the Earth's equator and return journeys to the Moon (Iorgulescu, 2018). CO_2_eq is expressed as the number of total emissions produced by an average EU household in one year (Ivanova & Wood, 2020), and in the equivalent of the surface area of melted Arctic ice the size of a tennis court (Notz & Stroeve, 2016).

The average distance travelled to Dubrovnik was almost twice the distance travelled to Bremen. With the travel-related emissions estimated for the Dubrovnik conference alone, 142 average EU households would have used up their entire emissions budget for one year, and the equivalent of 16 tennis courts of Arctic Sea ice would have melted.

Table 1 also shows average distances and emissions per attendee, online and on-site attendance, as well as conference locations. Independent t-tests confirmed that differences in emissions between online and on-site participation were significant during the hybrid conferences in Bratislava (*t*(525.01) = 12.42, *p* < .001, 95% CI [783.15, 1077.49]) and Bremen (*t*(728.05) = 11.10, *p* < .001, 95% CI[647.22, 925.44], see Table 1 for Ms and SDs).

On average, online participants saved 97.3% of emissions in Bratislava and 96.9% of emissions in Bremen compared to on-site participants.

Controlling for differences in distance between affiliation and conference venues in an ANCOVA (*F*(1, 2345) = 8286.00, *p* < .001, partial *η*^2^ = .78), we still find that the last two hybrid conferences produced significantly less travel-related emissions than the last on-site conference in Dubrovnik (*F*(2, 2345) = 68.35, *p* < .001, partial *η*^2^ = .06) (an additional ANCOVA with dummies for Bratislava and Bremen finds significant effects of Bratislava vs. Dubrovnik, and *F*(1, 2344) = 136.67, *p* < .001, partial *η*^2^ = .06 and for Bremen vs. Dubrovnik *F*(1, 2344) = 28.92, *p* < .001, partial *η*^2^ = .01). Thus, hybrid EHPS conferences result in fewer travel-related emissions than an on-site only conference.

**Figure 2.**
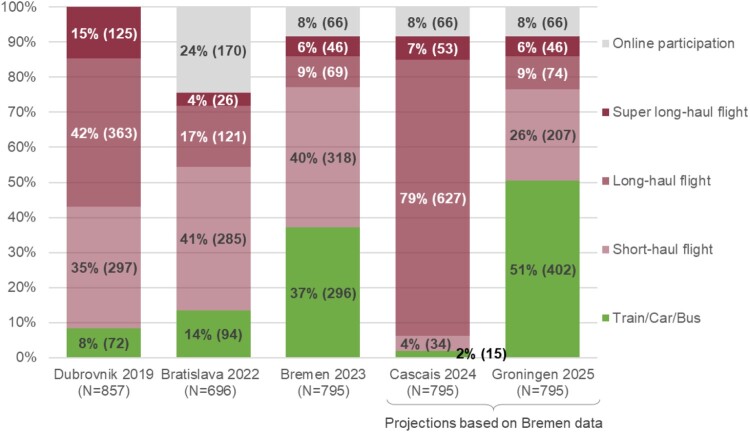
Estimated proportions (absolute numbers) of different modes of transport used to travel to EHPS conferences between 2019 and 2025. Note: Mode of transport estimated according to delegates’ affiliations and distances to conference venues.

Figure 2 shows that the location of conferences within Europe determines the estimated transport modes used by on-site participants. For venues in northern Europe, e.g. Bremen or Groningen, significantly more participants are able to travel by train, car or bus compared to Dubrovnik, Bratislava and Cascais (in green, *X^2^*(3938, 4) = 797.99; *p* < .001; CC = .410, *p* < .001; Cramer's V = .450, *p* < .001). The conference in Cascais is estimated to be accessible by ground travel for only 2% of all participants. Only the conference in Dubrovnik emitted slightly (but not significantly) more than Cascais, with fewer long-haul flights, but about 15% super-long-haul flights – estimated to be particularly emitting (e.g. because of flight altitude). An ANOVA shows significant differences of travel emissions for on-site participants between Dubrovnik, Bratislava and Bremen, Welch’s *F*(2, 1347.51) = 36.87, *p* < .001, partial *η*^2^ = .04. Post hoc comparisons using the Tukey HSD test indicate that mean emissions between Bremen and Bratislava did not differ significantly, but that Dubrovnik had significantly higher emission than the other two. If the projected means of Groningen and Cascais are added to this comparison, significant differences emerge, too (Welch’s *F*(4, 1736.01) = 32.27, *p* < .001, partial *η*^2^ = .04), with Dubrovnik and Cascais having comparable but significantly higher mean emissions than Bremen, Bratislava and Groningen (which did not differ).

**Figure 3.**
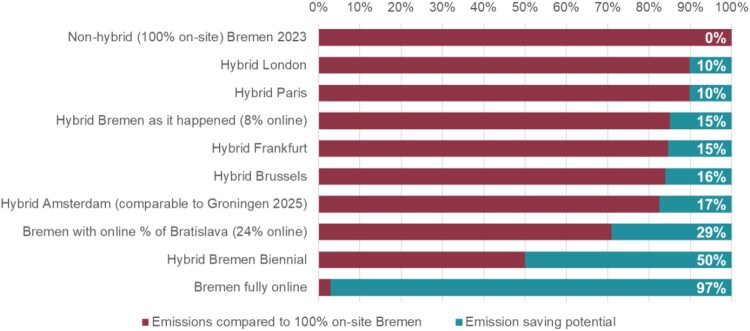
CO_2_eq emissions saving potentials by format and (hypothetical) EHPS conference location.

To estimate potential travel emissions savings, potential travel-related CO_2_eq emissions were calculated for five selected European cities, assuming the same number of participants as in Bremen (for detailed results see Table S3 in Supplement 1). Potential to save emissions by hybrid option and location is illustrated in Figure 3, assuming a hypothetical on-site only Bremen as baseline (non-hybrid option assuming travel emissions for the total number of participants).

Figure 3 shows that the hypothetical scenarios of fully online annual, or hybrid biennial conferences could have the greatest potential to reduce travel-related emissions. However, these options have been previously rejected by the EHPS community.

Selecting well-connected locations that would minimise travel distances for attendees, such as Amsterdam, could also reduce travel emissions. As the majority of participants travel from the UK, Germany and Netherlands, cities that minimise their travel distances have the highest saving potential (see Table S1 and S2 in Supplement 1, for attendee numbers by affiliation). Furthermore, increasing the online participation rate to the amount achieved in Bratislava (24%) has great saving potential, too.

## Discussion

The location of the EHPS conference substantially affects travel-related greenhouse gas emissions, and offering and promoting hybrid conferences helps reduce these emissions significantly. Given that on-site meetings are essential to the collegial atmosphere of the EHPS society, we suggest that the most travel emission savings can be achieved by these two approaches:

**1) *Offer attractive online participation during hybrid conferences to* create win-win-win situations for**
 - the EHPS – by including presentations by and discussions with scientists who would otherwise not be able to attend,
 - the scientists – by addressing inequalities in their ability to travel, funding, time availability, visa requirements, and by providing opportunities to balance daily demands with science (Cohen et al., 2020; Whitmarsh et al., 2020),
 - the climate – as online participation produces far fewer emissions than on-site participation.

The 24% of online participants at the 2023 EHPS in Bratislava saved 313 tonnes (29%) of emissions. In the post-conference survey, we asked delegates about their reasons for participating online (Heischkel et al., 2023):

 - 33% answered ‘obligations at home’,
 - 22% ‘cost’,
 - 14% ‘convenience’,
 - 5.5% ‘sustainability’ and
 - 17% had other reasons (e.g. health issues, war in Ukraine).

This shows that for some scientists, an online option is preferable.

To ensure an enjoyable and equitable online experience, the option for online participation should be announced prior to the conference and actively promoted, while guaranteeing the active participation of online attendees in discussions during the event. In light of the costs, a potential solution could be to enable passive online participation in rooms where all presentations are conducted on-site (e.g. questions only via chat). A few conference rooms could be equipped for remote participation, allowing online attendees to present live and interact with the on-site audience through video calls (for better online experiences see e.g. Moss et al., 2021).

**2) Choose venues that minimise travel-distances**

Locations such as Groningen, Frankfurt, Brussels and Amsterdam have the most potential to reduce travel emissions for EHPS conferences by optimising travel distances for most participants. Cities with good train connections should be chosen to facilitate ground travel in reasonable hours (while keeping problems such as over tourism and heat in larger cities in mind).

### Limitations

Our estimated emissions only cover travel and online participation emissions. Although the travel estimates are very large, they are conservative (as they consider only direct flights and land routes between participants’ bases and conference venues, excluding local transport). Other factors such as catering (e.g. percentage of plant-based food, food waste), accommodation and venue selection (e.g. percentage of eco-certificates), were not taken into account (see e.g. Neugebauer et al., 2020 for total emissions caused by conferences).

Our estimates of travel-related CO_2_eq of on-site participation (between 0.72 and 1.66 tonnes per person) aligne with previous studies (Bousema et al., 2020; Desiere, 2016; Jäckle, 2019; Jäckle, 2022; Klöwer et al., 2020; Milford et al., 2021; Neugebauer et al., 2020; Stroud & Feeley, 2015; Van Ewijk & Hoekman, 2021; Wortzel et al., 2021). However, participants did not self-report their mode of transport. Also, we did not take into account individual carbon offsetting measures. Effectiveness and promises of offsetting schemes have been criticised from various perspectives (cf. Jones & Lewis, 2023; Probst et al., 2023). However, where flying is unavoidable, offsetting via credible schemes (e.g. Atmosfair) can be recommended, rather than to ignore the environmental impact of air travel entirely.

### What next?

80% of people around the world are calling on global leaders to ‘act now and act boldly to fight the climate crisis’ (United Nations Development Programme, 2024, p. 3). To remain credible as a scientific society also researching planetary health, we need to subject our own scientific activities to transformation (Attari et al., 2016). National institutions, ministries, international organisations, and scientific societies have published recommendations and aim for climate-neutral meetings (e.g. the IFMSA General Assembly in August 2018). We do not lack urgency or role models. We lack clear EHPS guidelines.

There are numerous factors to consider when selecting a conference location, from the available offers of potential host cities, scientific and economic benefits for the host regions, equity considerations, attractiveness, price, safety and environmental impact. This multi-criteria decision making is not easy. By quantifying the travel emissions associated with EHPS conferences and proposing viable alternatives, we aim to initiate discussions on how to respect planetary boundaries and a liveable planet for all, while maintaining the spirit of the EHPS. We therefore urge the EHPS executive committee and future conference organisers to give greater priority to the climate impact of our meetings.

## Supplementary Material

Supplement_1_The_Carbon_Footprint_of_Travelling_to_conferences_of_the_EHPS_in_press.pdf

Supplement2_EHPS_online_emissions_according_to_Faber_2021.xlsx

## Data Availability

Data is available upon request from the corresponding author lisa.warner@medicalschool-berlin.de. Data has not been uploaded to a repository as the affiliation data reveals the location of conference delegates at an anonymised but individual level. Delegates only agreed to the use of data at an aggregated level. Therefore, Supplement 1 does not show national groups with less than five delegates.

## Note

1 CO_2_ equivalent (CO_2_eq) is used to convert emissions of different greenhouse gases into the equivalent amount of carbon dioxide with the same global warming potential.

## Open Scholarship

This article has earned the Center for Open Science badge for Open Materials. The materials are openly accessible at https://osf.io/fyu5x/.
