# Supplementary material for: A little more conversation, a little more action, please: the carbon footprint of travelling to conferences of the European Health Psychology Society
Source: Health Psychol Behav Med. 2025 Jan 2;13(1):2447454. doi: 10.1080/21642850.2024.2447454 (PMC11703385; doi:10.1080/21642850.2024.2447454)
Supplement: Supplement_1_The_Carbon_Footprint_of_Travelling_to_conferences_of_the_EHPS_in_press.pdf [file RHPB_A_2447454_SM3753.pdf]

Supplement 1 to publication:

Warner, L. M., Fleiner, R., Sproesser, G., Green, J. A., Rehackova, L., Inauen, J., Araújo-Soares, V., Teran-Escobar, C. (in press). A little more conversation, a little more action, please: The carbon footprint of travelling to conferences of the European Health Psychology Society. *Health Psychology and Behavioral Medicine.*

Supplemental information on methods of estimation

Travel emissions for onsite participants were estimated based on the estimates published by Klöwer et al. (2020) and Desiere (2016) in three steps

1. The shortest possible distance (direct distance taking into account the sphere of the earth) between the departure city and the conference location was calculated using google maps and R scripts.
2. This distance determined the assumed mode of transport and the respective CO_2eq emission rates per kilometre according to the estimates by Klöwer et al. (2020), with the limits for train travel adjusted to European conditions. Due to the well-developed railway infrastructure, the threshold to switch from ground travel to air travel of 400 km assumed by Klöwer for the US was expanded to 600 km as previously assumed by Desiere (2016).
   - Distance < 600 km = car/train/bus/rail = 60 gCO_2eq/km,
   - distance between 600 -1,500 km = short-haul flight = 200 gCO_2eq/km,
   - distance between 1,500-8,000 km = long-haul flight = 250 gCO_2eq/km and
   - distance > 8,000 km = super long-haul flight = 300 gCO_2eq/km.
3. The distance to the conference venue for each on-site participation was multiplied by the above CO_2eq emission rates per selected mode of transport and multiplied by two for the round trip.

For example, participants from London were calculated to have a distance of 1,691km to Dubrovnik (EHPS conference venue 2019). For this distance, a long-haul flight was assumed, which was assigned 250gCO_2eq per kilometre (423kgCO_2eq) and multiplied by 2 for the roundtrip (846kgCO_2eq).

Supplemental information on travel-related CO_2eq estimates from previous studies

Our estimates between 0.72 and 1.66 tonnes per person of travel-related CO_2eq are in line with previous studies on other conferences with average CO_2eq per participant in tonnes:
Bousema et al. (2020) = 1.79
Desiere (2016) = 0.48
Jäckle et al. (2019) = 0.50 to 1.30
Jäckle et al. (2022) = 0.4 to 3.4
Klöwer et al. (2020) = 2.86
Milford et al. (2021) = 0.61
Neugebauer et al. (2020) = 0.47
Stroud & Feeley (2015) = 2.50 to 3.00
Van Ewijk & Hoekman (2021) = 1.50 to 1.80
Wortzel et al. (2021) = 1.19 to 1.61

Emissions due to virtual participation were estimated according to the values published by Faber (2021). The estimations were adapted as EHPS conferences are multi-day, international events, with approx. 15 organisers and a duration of 8 hours per day. Variables such as website views, search queries, and the use of monitors and table lamps were calculated based on participant

numbers. As the conference lasts 3.5 days, total daily emissions are multiplied accordingly. We assumed that participants would attend for either a minimum of two hours or a maximum of eight hours per day, with emissions calculated for both scenarios in Excel spreadsheets available from Faber (2021, see Supplement 2). This resulted in the minimum of 27 kgCO_2eq for the entire conference for participants attending 2 hours/day and a maximum of 45 kgCO_2eq for the entire conference for participants attending 8 hours/day. In a second step, R code was used to vary the values between these minimum and maximum emissions at random, generating individual total emissions in g/CO2-eq for each digital participant within this range.

References:

Bousema, T., Selvaraj, P., Djimde, A. A., Yakar, D., Hagedorn, B., Pratt, A., Barret, D., Whitfield, K., & Cohen, J. M. (2020). Reducing the carbon footprint of academic conferences: the example of the American Society of Tropical Medicine and Hygiene. *The American Journal of Tropical Medicine and Hygiene*, *103*(5), 1758. https://doi.org/10.4269/ajtmh.20-1013

Desiere, S. (2016). The carbon footprint of academic conferences: evidence from the 14th EAAE congress in Slovenia. *EuroChoices, 15*(2), 56-61. https://doi.org/10.1111/1746-692X.12106

Faber, G. (2021). A framework to estimate emissions from virtual conferences. *International Journal of Environmental Studies, 78*(4), 608-623. https://doi.org/10.1080/00207233.2020.1864190

Jäckle, S. (2019). WE have to change! The carbon footprint of ECPR general conferences and ways to reduce it. *European Political Science, 18*(4), 630-650. https://doi.org/10.1057/s41304-019-00220-6

Jäckle, S. (2022). The Carbon Footprint of Travelling to International Academic Conferences and Options to Minimise It. In: Bjørkdahl, K., Franco Duharte, A.S. (eds) Academic Flying and the Means of Communication. Palgrave Macmillan, Singapore. https://doi.org/10.1007/978-981-16-4911-0_2

Klöwer, M., Hopkins, D., Allen, M., & Higham, J. (2020). An analysis of ways to decarbonize conference travel after COVID-19. *Nature, 583*(7816), 356-359. https://doi.org/10.1038/d41586-020-02057-2

Milford, K., Rickard, M., Chua, M., Tomczyk, K., Gatley-Dewing, A., & Lorenzo, A. J. (2021). Medical conferences in the era of environmental conscientiousness and a global health crisis: The carbon footprint of presenter flights to pre-COVID pediatric urology conferences and a consideration of future options. *Journal of Pediatric Surgery*, *56*(8), 1312-1316. https://doi.org/10.1016/j.jpedsurg.2020.07.013

Neugebauer, S., Bolz, M., Mankaa, R., & Traverso, M. (2020). How sustainable are sustainability conferences? Comprehensive Life Cycle Assessment of an international conference series in Europe. *Journal of Cleaner Production, 242*, 118516. https://doi.org/10.1016/j.jclepro.2019.118516

Stroud, J. T., & Feeley, K. J. (2015). Responsible academia: Optimizing conference locations to minimize greenhouse gas emissions. *Ecography*, *38*(4), 402-404. https://doi.org/10.1111/ecog.01366

Van Ewijk, S., & Hoekman, P. (2021). Emission reduction potentials for academic conference travel. *Journal of Industrial Ecology, 25*(3), 778-788. https://doi.org/10.1111/jiec.13079

Wortzel, J. R., Stashevsky, A., Wortzel, J. D., Mark, B., Lewis, J., & Haase, E. (2021). Estimation of the carbon footprint associated with attendees of the American Psychiatric Association Annual Meeting. *JAMA Network Open*, *4*(1), e2035641. https://doi.org/10.1001/jamanetworkopen.2020.35641

Dubrovnik 2019			Bratislava 2022			Bremen 2023		
	N	%		N	%		N	%
United Kingdom	200	23.3%	United Kingdom	174	25.0%	Germany	185	23.3%
Germany	67	7.8%	Germany	72	10.3%	United Kingdom	153	19.2%
Netherlands	55	6.4%	Netherlands	58	8.3%	Netherlands	89	11.2%
United States	45	5.3%	Switzerland	40	5.7%	Ireland	38	4.8%
Croatia	43	5.0%	Ireland	35	5.0%	Poland	30	3.8%
France	43	5.0%	France	29	4.2%	Belgium	25	3.1%
Ireland	40	4.7%	United States	28	4.0%	Switzerland	25	3.1%
Switzerland	31	3.6%	Slovakia	26	3.7%	France	23	2.9%
Japan	29	3.4%	Belgium	23	3.3%	Australia	21	2.6%
Poland	28	3.3%	Australia	21	3.0%	Italy	19	2.4%
Australia	22	2.6%	Poland	17	2.4%	Portugal	17	2.1%
Thailand	22	2.6%	Italy	15	2.2%	Austria	16	2.0%
Canada	21	2.5%	Portugal	15	2.2%	Japan	14	1.8%
Portugal	18	2.1%	Canada	13	1.9%	Israel	13	1.6%
Slovakia	16	1.9%	Austria	10	1.4%	United States	12	1.5%
Finland	12	1.4%	Finland	10	1.4%	Finland	11	1.4%
Italy	12	1.4%	Romania	10	1.4%	Sweden	10	1.3%
Belgium	11	1.3%	Israel	9	1.3%	Canada	9	1.1%
Romania	10	1.2%	Croatia	8	1.1%	Spain	8	1.0%
Turkey	10	1.2%	Japan	7	1.0%	Slovakia	7	0.9%
Austria	9	1.1%	Spain	7	1.0%	Romania	6	0.8%
Bulgaria	9	1.1%	Lithuania	6	0.9%	New Zealand	5	0.6%
Israel	9	1.1%	Chile	5	0.7%	South Africa	5	0.6%
South Africa	9	1.1%	Hungary	5	0.7%	-	-	-
New Zealand	8	0.9%	-	-	-	-	-	-
Spain	8	0.9%	-	-	-	-	-	-
Hungary	7	0.8%	-	-	-	-	-	-
Lithuania	6	0.7%	-	-	-	-	-	-
Norway	6	0.7%	-	-	-	-	-	-
Cyprus	5	0.6%	-	-	-	-	-	-
Luxembourg	5	0.6%	-	-	-	-	-	-
Sweden	5	0.6%	-	-	-	-	-	-
+ 24 countries with n < 5			+ 30 countries with n < 5			+ 26 countries with n < 5		

Supplement 1, Table S1: Frequencies of affiliated country for participants of the past three EHPS conferences

Supplement 1, Table S2: Frequencies of affiliated country for participants of the past three EHPS conferences combined (sum of Dubrovnik 2019, Bratislava 2022, Bremen 2023)

Country	N	%
United Kingdom	527	22.4%
Germany	324	13.8%
Netherlands	202	8.6%
Ireland	113	4.8%
Switzerland	96	4.1%
France	95	4.0%
United States	85	3.6%
Poland	75	3.2%
Australia	64	2.7%
Belgium	59	2.5%
Croatia	53	2.3%
Japan	50	2.1%
Portugal	50	2.1%
Slovakia	49	2.1%
Italy	46	2.0%
Canada	43	1.8%
Austria	35	1.5%
Finland	33	1.4%
Israel	31	1.3%
Romania	26	1.1%
Spain	23	1.0%
Thailand	22	0.9%
Sweden	18	0.8%
Hungary	16	0.7%
Lithuania	16	0.7%
South Africa	16	0.7%
New Zealand	15	0.6%
Bulgaria	14	0.6%
Turkey	13	0.6%
Cyprus	11	0.5%
Greece	9	0.4%
Norway	9	0.4%
China	7	0.3%
Latvia	7	0.3%
Singapore	7	0.3%
Chile	6	0.3%
Luxembourg	6	0.3%
Russia	6	0.3%
South Korea	6	0.3%
Czechia	5	0.2%
+ 31 countries with n < 5		

Supplement 1, Table S3: Estimates of CO_2eq emissions per participant and potential conference location by mode of participation (in-person only travel emission / online only IT emissions)

	Amsterdam			Brussels			Frankfurt			London			Paris		
	hybrid (on site + online)	on site only	online only	hybrid (on site + online)	on site only	online only	hybrid (on site + online)	on site only	online only	hybrid (on site + online)	on site only	online only	hybrid (on site + online)	on site only	online only
N of participants registered	795	729	66	795	729	66	795	729	66	795	729	66	795	729	66
n from Europe (%)	697 (87.7%)	645 (88.5%)	52 (78.8%)	697 (87.7%)	645 (88.5%)	52 (78.8%)	697 (87.7%)	645 (88.5%)	52 (78.8%)	697 (87.7%)	645 (88.5%)	52 (78.8%)	697 (87.7%)	645 (88.5%)	52 (78.8%)
Total km travelled by all participants	-	2,336,090	-	-	2,360,927	-	-	2,389,216	-	-	2,497,901	-	-	2,535,784	-
M (*SD*) in km roundtrip per participant	-	3,205 (6,479)	-	-	3,238 (6,514)	-	-	3,277 (6,421)	-	-	3,426 (6,543)	-	-	3,478 (6,525)	-
Number of return trips to the moon	-	6.08	-	-	6.14	-	-	6.22	-	-	6.50	-	-	6.60	-
Number of trips around earth	-	58.29	-	-	58.91	-	-	59.62	-	-	62.33	-	-	63.28	-
Total CO2eq in t for conference	576	574	1,71	586	584	1.64	591	589	1.70	627	625	1.69	626	624	1.68
M (*SD*) CO2eq in t per participant	0.72 (1.88)	0.79 (1.95)	0.03 (0.01)	0.74 (1.89)	0.80 (1.96)	0.02 (0.01)	0.74 (1.87)	0.81 (1.94)	0.03 (0.01)	0.79 (1.91)	0.86 (1.98)	0.03 (0.01)	0.79 (1,91)	0.86 (1.98)	0.03 (0.01)
CO2eq in # of av. emissions of an EU household per year	57.61	57,44	0.17	58.61	58.45	0.16	59.08	58.91	0.17	62.70	62.53	0.17	62.65	62.48	0.17
CO2eq in tennis courts of melted artic sea ice	6.62	6.61	0.02	6.74	6.72	0.02	6.79	6.77	0.02	7.21	7.19	0.02	7.20	7.18	0.02
